# Biomedical Informatics for Computer-Aided Decision Support Systems: A Survey

**DOI:** 10.1155/2013/769639

**Published:** 2013-02-04

**Authors:** Ashwin Belle, Mark A. Kon, Kayvan Najarian

**Affiliations:** ^1^Department of Computer Science, Virginia Commonwealth University, Richmond, VA 23284, USA; ^2^Department of Mathematics and Statistics, Boston University, Boston, MA 02215, USA

## Abstract

The volumes of current patient data as well as their complexity make clinical decision making more challenging than ever for physicians and other care givers. This situation calls for the use of biomedical informatics methods to process data and form recommendations and/or predictions to assist such decision makers. The design, implementation, and use of biomedical informatics systems in the form of computer-aided decision support have become essential and widely used over the last two decades. This paper provides a brief review of such systems, their application protocols and methodologies, and the future challenges and directions they suggest.

## 1. Introduction

Over the past several decades the uses and applications of biomedical informatics for computer-aided medical diagnostics and decision support systems have become ubiquitous in clinical settings. Adaptations of decision support systems powered by biomedical informatics in either complex or simple forms were seen as early as the 1970s. A 1994 survey [1] indicates that the literature relevant to this field dates back to as early as the mid-1950s. 

With advances in technologies related to medical signal and image acquisition, it can be seen that there has been an escalation of complexity in collected medical data. Apart from medical data being inherently more complex, the sheer volume of such data collected per patient is growing rapidly. Currently medical devices and high-throughput measurement systems produce thousands of images and large volumes of other data per patient in seconds, making it difficult for physicians to parse through the information while providing timely diagnoses and prognoses. There is a present significant need for development and improvement of computer-aided decision support systems in medicine, with an expected amplification in the future. 

Clinical implementations of biomedical informatics methods in the form of computer-based decision support systems were seen as early as 1971, when Dombal's system AAPhelp, developed at Leeds University, attempted to automate the diagnosis of acute abdominal pain [2]. In 1974 a system called INTERNIST-I [3], a rule-based expert system designed to aid the diagnosis of complex medical problems in internal medicine, was developed. These represent prominent developments in early implementations of biomedical informatics systems, among many computer-based diagnostic decision support systems. Since their inception there has been a substantial evolution, with wide acknowledgment of their success in improving practitioners' performance and patient outcomes. 

With broad research conducted in the area, there have been review studies on related topics. A book by Greenes [4] outlines general concepts and future directions for clinical decision support systems. Similarly, an article by Madabhushi et al. [5] describes development of computer-aided prognosis systems for predicting patient and disease outcomes using multiscale, multimodal medical data. Miller's article in 1994 provides a comprehensive list of important work conducted on diagnosis and decision support between 1954 and 1993 [1]. Similarly, a more recent article by Pearson et al. provides a systematic review of computerized clinical decision support systems between 1990 and 2007 [6]. In this work 56 different studies are considered, of which 38 are on systems used in therapy initiation, 23 involve computer-based monitoring of patients during therapy, and three study conditions for termination of therapy. From their outcomes, the authors infer that the most consistently effective computer-based systems are those that initiate advice to fine-tune existing therapies by improving patient safety, adjusting the doses, durations forms of prescribed drugs, or increasing the laboratory testing rates for patients on long-term therapies.

Some previous studies also provide insights into more specific subgenres of biomedical informatics methods and their implementations in the form of computer-aided diagnosis systems. Tourassi discusses systems that provide diagnostic interpretations based on image texture analysis [7] and Stivaros et al. [8] focus on the impacts of decision support systems in clinical radiological practice. 

This paper provides a general survey of applications and methodologies in biomedical informatics that have been implemented as computer-aided decision support systems and discusses the resulting challenges, for example, in validation of such systems, and in adoption levels among end users. The paper is organized as follows. some major application areas for the above-mentioned systems are described, followed by a discussion of important methodologies employed. Then there is a brief look at the validation and success criteria for these systems, followed by the conclusion and discussion of future directions. 

## 2. Applications

There are a number of application areas medicine for which computer-aided decision support systems have become designed and implemented. Some of the major application areas are discussed below.

### 2.1. Radiology

Here, computer-based image processing and analysis have been an active research area. Combining visualization, image processing, and machine learning for decision-making has provided an added advantage for clinical applications. With multiple technologies for medical imaging such as computed tomography (CT), X-rays, magnetic resonance imaging (MRI), and functional MRI (FMRI), numerous biomedical informatics methods have been designed for application-specific solutions. A study by Van Ginneken et al. surveys over 150 publications before 2001 on computer-aided diagnosis in chest radiography [9]. This survey emphasizes the continued interest in computer-aided diagnosis for chest radiography. There are also several studies on developing decision-making systems using automated analysis of CT scans. These include Chen et al.'s [10, 11] study, which focuses on developing a computer aided diagnostic system that automatically analyses brain CT scans of patients with traumatic brain injury (TBI). The system also automatically estimates the level of the intracranial pressure (ICP) within the brain. Another study by Davaluri et al. discusses the development of computer-assisted decision-making systems for pelvic injuries [12]. Wu et al. focus on fracture detection in traumatic pelvic injury patients and discusses an automated method for quantifying the size of fractures from CT images of patients with pelvic injuries [13]. Stivaros et al. provide an overview of underlying design and functionality of radiological decision support systems, with supporting examples of the development and evolution of such systems in the past 40 years [8].

### 2.2. Emergency Medicine and Intensive Care Units

One of the most active areas of research in the realm of biomedical informatics and decision support systems is emergency medicine. For patients in intensive care units (ICU) and emergency rooms, it is critical that diagnosis and treatment are provided in a timely manner. Since critical care units typically experience a heavy strain on resources, it becomes important to manage and dispense resources to critically ill patients who need it the most. Computer-aided decision support systems play a vital role in reducing diagnosis time, improving resource allocation efficiency, and decreasing patient mortality. Ji et al. describe a study that provides a comparative analysis of computer-assisted decision-making systems for traumatic injuries [14]. Systems such as one developed by Frixea et al. show how case-based reasoning techniques for the estimation of patient outcomes and resource utilizations can improve patient care dramatically in ICUs [15]. Kumar et al.'s study [16] presents a clinical decision support system which combines both case-based reasoning and rule-based reasoning and that performs well with real and simulated ICU data. Raschke et al. describe a computer alert system which is designed to recognize averse drug events (AEDs) in hospital settings [17]. This system is reported to be capable of generating alerts for patients with increased risk of AEDs. The study states that during the 6-month trial of the system, a total of 265 (44%) of the 596 true positive alerts were unrecognized by the physicians prior to the alert notification, hence showing a great promise for applications in continuous patient monitoring. 

### 2.3. Cardiovascular Medicine

Having continuous or interventional monitoring of cardiovascular signals for diagnosing ailments or predicting impending cardiac events can be an extremely useful tool. Currently there are several research biomedical informatics studies attempting to develop computer-aided solutions for various aspects of cardiovascular medicine. A study conducted by Polat et al. describes a computer-aided diagnosis system that automatically identifies and classifies arrhythmia from the analysis of patients' electrocardiograph (ECG) signals [18]. The authors claim 100% accuracy in classification within the dataset used. Watrous reviews various studies which use auscultation signal of the heart for analysis and provide diagnostics decision support to physicians [19, 20]. Shandilya et al. present their work on the design and development of a nonlinear method for analysis of ventricular fibrillation using ECG signals to predict high yields accuracy for defibrillation success [21]. The study also describes the incorporation of PetCO2 signal to noticeably increase the predictive models robustness.

### 2.4. Dental Applications

Computerized clinical diagnosis and decision support systems have also seen much success in the field of dentistry. Firestone et al. describe a clinical decision support system on observer performance which was a knowledge-based system performing image analysis on radiographic images [22]. This study involved 102 approximal surface radiographic images and sixteen general practitioners for identifying the presence of caries and whether restoration was required. The paper states that those dental practitioners who used the system to produce their diagnoses showed significant increases in their ability to diagnose caries correctly, with an increased overall diagnostic accuracy and recommendation for restoration of detected cavitated surfaces. Similarly, Olsen et al. propose a computer-aided caries detection system using image analysis of data from intraoral cameras [23]. This paper describes a feasibility study of using advanced image processing and machine learning techniques to identify caries from digital images. 

### 2.5. Cancer

Biomedical informatics has begun to play an important role in cancer detection and treatment. In a study conducted by Lisboa and Taktak, a systematic review of several studies involving decision-making tools in the field of cancer is presented [24]. In particular, the review focuses on those studies that apply artificial neural network methods. Using 27 studies which were either clinical trials or randomized controlled trials, the paper reports that 21 of those studies show benefits in treatment while the remaining 6 did not. Another study by Jesneck discusses an approach to optimize computer-aided decision-making for cancer diagnosis by combining heterogeneous information from different modalities [25]. The authors claim that their proposed method at times outperforms two popular machine-learning techniques, that is, linear discriminant analysis and artificial neural networks. A study by Madabhushi et al. briefly discusses four different computer-aided support systems for cancer diagnosis and prognosis [26]. The first system is an image-based risk score algorithm for predicting the outcome of the estrogen receptor marker for breast cancer patients based on digitized biopsy. The second system is discussed in the paper segments and determines the extent of lymphocytic infiltration from digitized histopathology. The third method described distinguishes patients with varying Gleason grades of prostate cancer, from needle biopsy specimens. The final system integrates quantitative image features extracted from digitized histopathology with protein expression measurements obtained from mass spectrometry, in order to distinguish between low and high risk patients with prostate cancer recurrence following radical prostatectomy. Jiang et al. published a paper evaluating the reduction of interobserver variability in the interpretation of mammograms while using computer-aided diagnosis tools [27]. The authors state that using computer-aided diagnosis tools has the potential to reduce variability amongst expert opinions as well as improve diagnostic accuracy for the interpretation of mammograms. Similarly, another study by Cheng et al. summarizes and compares the methods used in various enhancement and segmentation algorithms, mammographic feature extraction, classifiers, and their performances for detection and classification of microcalcification clusters [28]. A paper by Mazurowski et al. describes an optimization framework for improving case-based computer-aided decision systems used for screening mammography [29]. The paper claims that the proposed method significantly improves the overall performance and breast mass detection rates of such systems. Cai et al.'s paper describes a study based on classification of cancer subtypes and survival prediction in diffuse large B-cell lymphoma (DLBCL) using levels of genes [30]. Research by Rangayyan et al. describes refined methodologies that have been developed in computer-aided breast cancer diagnostic systems [31]. The research presents new detection techniques for identifying subtle signs of breast cancer addressing difficult problems such as focal architecture distortion and global bilateral asymmetry. 

### 2.6. Pediatric Medicine

Computer-aided diagnosis and decision support systems have become popular for a variety of applications in neonatal and pediatric care units. A study by Ramnarayan et al. discusses the potential of diagnostic and decision support systems in pediatric settings with a case study of a web-based pediatric differential diagnostic tool [32]. Ramnarayan also explains the various usages of such diagnostic aid systems and outlines its future direction for research in another article [33]. Frizea et al. discuss an artificial intelligence-based system which uses case-based reasoning for estimating medical outcomes and resource utilization. The paper explains how such a system was initially intended for adult ICU care units and then was modified to function in neonatal ICUs. The paper reports that the results from a short clinical pilot study performed in neonatal ICU were very encouraging and captured the interests of physicians for their potential clinical usefulness. Tan et al. published a review paper on clinical decision support systems for neonatal care [34]. The objective of this review was to find whether the use of clinical decision support systems had any effect on the mortality and morbidity rate of newborn infants, and to see if there was any change in the performance of the physicians treating these infants. Mack et al. also published a similar review study of decision support systems available in pediatric intensive care units [35]. The paper provides a look into the factors that are involved in the applications of such systems in pediatric practices, including liability, human factors, audit trails, engineering, and alert fatigue. The paper concludes that selecting and implementing such systems in clinical practice requires a great deal of caution, though when done correctly it has good potential for benefiting and improving clinical practice in pediatric intensive care units. 

## 3. Methodology

There are several fundamental computational methodologies used toward developing these biomedical informatics and computer-aided diagnosis support systems. The types of techniques and methods are based on application areas and required performance metrics. Some of the major aspects of such systems are discussed below.

### 3.1. Expert Systems, Case-Based Reasoning, and Rule-Based Systems

Methods such as rule-based systems (fuzzy and crisp), expert systems, and case-based reasoning are formed from the knowledge accumulated from experts of a given field. Opinions, diagnoses, and prognoses, among other components, are compiled to form rule-based analysis structures, based on which specific concepts for diagnosis solutions are developed. Kumar et al. present a hybrid decision support system which was designed based on both case-based and rule-based reasoning [16], which is applied to ICU facilities for aiding physicians in decision making. Another study by Innocent describes an approach to computer-aided medical diagnosis systems for clinical contexts using fuzzy logic [36]. In this system, knowledge from experts is compiled into fuzzy cognitive maps and logical structures to estimate a stage of disease using temporal information in symptom durations.

### 3.2. Signal and Image Processing

Some computerized diagnostic aid systems use a variety of patient data for analysis in developing diagnostic suggestions. These systems analyze raw patient signals and images to extract useful features and trends based on which diagnostic and decision support information is computed and presented to physicians. For instance, Polat et al. describe a signal processing system that analyzes ECG to classify cases of arrhythmia in diseased persons [18]. The signal processing system, developed by Shandillya et al., detects the ideal time to defibrillate patients undergoing cardiac arrest or ventricular fibrillation [21, 37]. Davaluri et al. proposes an image processing system which uses CT images of patients with pelvic injuries to produce a quantitative and qualitative assessment of detected hemorrhaging [38]. Similarly, Wu's work on developing a computer-assisted fracture detection system automatically processes several CT slices of pelvic injury patients to identify and quantify potential fractures [39]. 

### 3.3. Machine Learning

Due to the continuous advancements in the field of machine learning, more complex and sophisticated biomedical informatics systems are being designed. Systems that have the ability to predict and classify diseases fundamentally rely on some type of machine learning methodology. There is no one superior machine learning technique that can be applied toward all learning problems; instead the best method depends on the type of application. For example, Lisboa's study provides a systematic review of neural networks in decision support systems for cancer diagnosis and treatment [24]. Jesneck et al. describe how a customized machine learning technique outperforms standard techniques such as artificial neural networks and linear discriminant analysis in their study using cancer datasets [25]. Ji et al. compare a variety of machine learning techniques used in decision-making systems for traumatic injury assessment [40]. 

## 4. Impact of Computer-Aided Decisions in Bioinformatics 

In the last two decades, bioinformatics has emerged as a vibrant and rapidly growing field. However, as shown above, the majority of computer-aided decision support systems is implementations of biomedical informatics systems, so that very few of the currently used computer-aided support systems are based on bioinformatics approaches, which is understandable given the age of the field.

A study by Maojo et al. provides a comparison of histories, fundamental foundations, and scientific approaches of the two complementary yet separate fields, that is, of medical informatics and bioinformatics [41]. With most computerized clinical diagnostic aids being developed under the umbrella of biomedical informatics, Maojo et al. explain how inclusion of knowledge from bioinformatics can strengthen applications development for healthcare. The authors emphasize that future research designed as a hybrid of both informatics subdisciplines is the key to making significant advances in clinical practice and biomedical research. 

The effort to combine multimodal data and to combine biomedical informatics and bioinformatics has already shown a great promise. As mentioned, Madabhushi et al. describe research on computer-aided prognosis and diagnosis systems using multi-modal data fusion, including computerized image analysis and digitized patient data such as tissue and genomic information for predicting outcomes and survival [5]. These projects use protein expression and other data, processed by typical biomedical informatics methods, to diagnose and develop prognoses for cancer cases. Huang et al. analyzed and published a time series microarray gene expression profiles dataset to predict how patients respond to pegylated interferon treatment [42, 43]. Computer-aided decision systems adapted with bioinformatics knowledge have begun to show positive impact on virological research. For instance a paper by Huang et al. describes a computational method in identifying the underlying mechanisms for HIV-1 resistance in some people based on gene expression profiles and the analysis of the network of virus-host interaction [44]. Similarly, another study describes a novel approach in diagnosing liver cirrhosis and hepatocellular diseases using a network based analysis [45].

## 5. Validation and Criteria for Success

With numerous clinical implementations of decision support systems for a variety of medical applications, it is essential to have a systematic method to verify, validate, and compare different systems and their performances. For instance, Berner et al. compare the performance of four computer-based diagnostic systems applied towards internal medicine applications, namely: Dxplain, Iliad, Meditel, and QMR [46]. These systems have all been noted in various publications in their phases of development, evaluation, and applications [47, 48]. The authors have tested these systems on identical diagnostically challenging cases and measured the performances of each of these systems on several developed measurement scales. Estimates of performance were provided with a prospectively determined set of test specifications, using cases with a range of content and difficulty. Another study by Manotti et al. assesses the performance of another decision support system pertinent to oral anticoagulant treatment [49]. In this paper the authors describe a clinical trial of the system with several patients across multiple clinics, to test whether the computer-based decision support system is efficient in stabilizing patients undergoing oral anticoagulant treatment by initiating and maintaining therapy. With statistical analysis of performance measures the paper reports that the decision support system improves the quality of anticoagulant treatment, both during long-term treatments and in early, unstable phases of treatment. 

Several publications also explain the various criteria that need to be considered for successful development and application of a computer-assisted decision support system. Along these lines, Kaplan reviews the literature related to clinical decision support systems with an emphasis on evaluation criteria [50]. In the paper the author explains that with the success seen so far there is a general enthusiasm amongst physicians and researchers with the potential of computerized clinical decision support systems to improve the quality of healthcare. Nonetheless, there is a lack of theoretical understanding especially from a nonphysician's perspective of such systems and also as to why certain diagnostic aid systems may not be effective. Similarly Dreiseitl and Binder consider the effects of decision support systems on physicians' opinions, in particular to see whether they, doctors, value its opinion when it contradicts theirs [50]. They conclude that physicians are fairly susceptible to accepting recommendations of such decision support systems, making quality assurance and validation of more paramount importance. Ramnarayan et al. highlight the importance of developing a reliable and valid composite scoring system to measure the impact of diagnostic decision support on the quality of healthcare [32]. They claim that the scoring systems they describe can be further used in assessing outcome measures of other study types, involving computer-assisted diagnostic systems. Song et al. discuss the various approaches, goals, and characteristics of computer-aided healthcare workflows [51]. The authors analyze the workflow application issues and software challenges in the perspective of medical informatics and software engineering. Niès et al. published a paper listing four key characteristics pertaining to the content of diagnosis support that are associated with the success of computerized clinical decision support systems [52]. The paper provides a systematic review of published trials to identify the characteristics of the adopted methodologies and technicalities of those studies that assess the efficacy of clinical decision support systems. 

## 6. Conclusion and Future Directions

Table 1 describes the overall strengths and weakness of existing computer-aided decision support systems, and research in some of the application areas discussed in this paper. 

With the sheer number of biomedical informatics methods implemented as computer-assisted diagnosis and decision support systems, along with the vast amount of research in this field, such systems are inevitably becoming an inherent part of medicine. The systems are becoming capable of solving more complex and sophisticated clinical problems. By establishing systematic processes for validation and verification, these computer-aided systems can become much more reliable and thereby improve quality of diagnostic decisions, as well as reduce variance among physicians' opinions. The unique capabilities of these systems allow care givers and researchers to gain insight into current clinical issues in ways that would have been impossible in the past. 

Furthermore, it is becoming advantageous to fuse information derived from medical data with multiple modalities to provide more robust diagnoses and treatment plan suggestions [5, 10, 40]. The current fusion of biomedical informatics and bioinformatics techniques will accelerate the formation of a new generation of system-biologic computer-aided decision support systems, that will process and combine information in molecular data, signals and images, and demographics, among others. These and many other sources of patient data will allow such systems to form much more specific and personalized recommendations.

Applying advances in computational methods and techniques towards such systems can help in problems such as overfitting of outputs towards specific types of data, susceptibility to incomplete/missing data, and presence of conflicting information from different sources. These advances in the computational methods can also improve the quality of information accessed from feature extraction and feature selection—this improvement is often a critical step prior to classification and/or clustering. 

While computerized diagnostic and prognostic decision support systems have proved to be instrumental in medicine, it appears that an even more significant contribution of these systems can be expected when they further evolve to process and integrate newer and even broader types of patient data. 

## Figures and Tables

**Table 1:** Stregnths and weaknesses of existing computer-aided decision support systems and research in different application areas.

Application areas	Strengths	Weaknesses
Cancer	(i) An abundance of molecular assays and data are available for many cancer cases; these can be used towards developing strong decision support systems	(i) More should be done to integrate knowledge from molecular-based and image-based sources available for cancer detection (ii) There is a need to develop better schemes and methods for validating the effectiveness of the existing and upcoming systems in this area

Radiology	(i) A variety of effective computational techniques exists for many applications in radiology (ii) It is one of the fastest growing fields using applications of computer-aided decision systems	(i) Most of the research in this area suffers from lack of comprehensive datasets (ii) Most of these studies do not include knowledge of illness/injury/complication into the decision-making process

Emergency medicine	(i) Although there are only a few systems that have been adopted into clinical practices, the existing systems have shown a positive impact on the cost and quality of healthcare (ii) There is a significant potential for computer-aided systems in this area since emergency medicine and trauma are very time and resource critical aspects of healthcare	(i) Accuracies of existing systems may not be sufficient for clinical uses (ii) A variety of illnesses and injuries have not yet been addressed by computer-aided decision support systems (iii) There is a lack of comprehensive validation of the short-/long-term impacts on these systems using sufficiently large datasets

Cardiovascular medicine	(i) Since heart disease is among the leading causes of death, computer-aided decision systems here have potentially very high impact on world health (ii) While most cardiovascular-based intelligent decision support systems suffer from high false positives, they often help detect disease at early stages	(i) These systems usually incorporate only a portion of available patient information. More variety in information sources may be required in the decision-making process to reduce false positives (ii) There is a lack of a comprehensive validation process. Existing research claims need to be tested in more real-world settings

Dental	(i) Existing systems have shown capability for detecting dental complications at early stages (ii) Such early detection facilitates better practice of preventive care	(i) Some of the technologies used for capturing the information for computer-aided decision support systems are relatively expensive and hence preventing them from being widely adopted in practice
